# *SlGT11* controls floral organ patterning and floral determinacy in tomato

**DOI:** 10.1186/s12870-020-02760-2

**Published:** 2020-12-14

**Authors:** Liling Yang, Shilian Qi, Arfa Touqeer, Haiyang Li, Xiaolan Zhang, Xiaofeng Liu, Shuang Wu

**Affiliations:** 1 College of Horticulture, FAFU-UCR Joint Center and Fujian Provincial Key Laboratory of Haixia Applied Plant Systems Biology, Fujian Agriculture and Forestry University, Fuzhou, China; 2 State Key Laboratories of Agrobiotechnology, Beijing Key Laboratory of Growth and Developmental Regulation for Protected Vegetable Crops, MOE Joint Laboratory for International Cooperation in Crop Molecular Breeding, China Agricultural University, Beijing, China

**Keywords:** Tomato, SlGT11, Floral reversion, Floral organ identity, Floral determinacy

## Abstract

**Background:**

Flower development directly affects fruit production in tomato. Despite the framework mediated by ABC genes have been established in Arabidopsis, the spatiotemporal precision of floral development in tomato has not been well examined.

**Results:**

Here, we analyzed a novel tomato *stamenless like flower* (*slf*) mutant in which the development of stamens and carpels is disturbed, with carpelloid structure formed in the third whorl and ectopic formation of floral and shoot apical meristem in the fourth whorl. Using bulked segregant analysis (BSA), we assigned the causal mutation to the gene *Solanum lycopersicum GT11* (*SlGT11*) that encodes a transcription factor belonging to Trihelix gene family. *SlGT11* is expressed in the early stages of the flower and the expression becomes more specific to the primordium position corresponding to stamens and carpels in later stages of the floral development. Further RNAi silencing of *SlGT11* verifies the defective phenotypes of the *slf* mutant. The carpelloid stamen in *slf* mutant indicates that SlGT11 is required for B-function activity in the third whorl. The failed termination of floral meristem and the occurrence of floral reversion in *slf* indicate that part of the C-function requires SlGT11 activity in the fourth whorl. Furthermore, we find that at higher temperature, the defects of *slf* mutant are substantially enhanced, with petals transformed into sepals, all stamens disappeared, and the frequency of ectopic shoot/floral meristem in fourth whorl increased, indicating that SlGT11 functions in the development of the three inner floral whorls. Consistent with the observed phenotypes, it was found that B, C and an E-type MADS-box genes were in part down regulated in *slf* mutants.

**Conclusions:**

Together with the spatiotemporal expression pattern, we suggest that *SlGT11* functions in floral organ patterning and maintenance of floral determinacy in tomato.

**Supplementary Information:**

The online version contains supplementary material available at 10.1186/s12870-020-02760-2.

## Background

Flowers of angiosperms are the reproductive organs playing an important role in reproduction. A typical eudicot flower, such as *Arabidopsis* and tomato, consists of four different organs arranged in four whorls at the tip of floral shoot. Based on genetics studies on model plants including *Arabidopsis* [1–3], *Antirrhinum majus* [1] and *Petunia hybrid* [4], an elegant model involving ABCDE class genes, has been proposed to explain the organ patterning in flower. In *Arabidopsis*, A class genes *APETALA1* (*AP1*) and *APETALA2* (*AP2*) are involved in the development of sepals and petals. B class genes *APETALA3* (*AP3*) and *PISTILLATA* (*PI*) can form protein complexes with C class gene *AGAMOUS* (*AG*) and E class genes *SEPALLATAs* (*SEPs*) to promote stamens development. The carpels formation is regulated by both C class genes *AGs* and E class genes *SEPs*. The interference of ABCDE genes leads to confusion in the identity of floral organs [3, 5]. Compared with *Arabidopsis*, tomato genome has more homologous ABCDE genes. Tomato possesses four B class homologous genes, two DEF lineage genes-*Tomato APETALA3* (*TAP3*) and *Tomato MADS-box 6* (*TM6*), two GLO genes-*Solanum lycopersicum GLOBOSA* (*TPIB, SlGLO1*) and *Tomato PISTILLATA* (*TPI, SlGLO2*) [6, 7]. In tomato, there are two C class homologous genes (*TOMATO AGAMOUS 1* (*TAG1*) and *TOMATO AGAMOUS-LIKE 1* (*TAGL1*)) [8, 9] and six E class homologous genes (*Tomato MADS-box 5* (*TM5*), *TM29*, *JOINTLESS-2* (*J2*), *ENHANCER-of-JOINTLESS2* (*EJ2*), *RIPENING INHIBITOR* (*RIN*) and *Solyc04g005320*) [10]. Although the ABC genes clearly have similar functions between *Arabidopsis* and tomato, they may have separate functions independent of each other.

The development of stamens and carpels has drawn particular attention, as the regulation of these two floral parts is important for crop breeding. In *Arabidopsis*, mutations in the B-class genes *APETALA3* (*AP3*) or *PISTILLATA* (*PI*) promote the conversions of petals into sepals and stamens into carpels [1]. Similarly, the tomato *stamenless* mutant was identified to have mutations in B class gene *TAP3* [11, 12]. In the mutant, stamens are completely transformed into carpels which are fused with the carpels in the fourth whorl to form a unique gynoecium, and petals are partially transformed into sepals [11, 12]. The silencing of another B class gene *TM6* also produced similar phenotypes [6, 13]. Mutants of the B-class genes *DEFICIENS* (*def*) and *GLOBOSA* (*glo*) triggered the same homeotic transformations in *Antirrhinum* [14]. The rice *stamenless 1*(*sl1*) mutant exhibits homeotic conversions of lodicules and stamens to palea/lemma like organs and carpels, which resembles the mutant of B class gene *SPW1* [15]. Another type of homeotic transformation has also been reported in these species. In *Arabidopsis*, the mutant of the C-class gene *AG* showed that the stamens and carpels were transformed into petals and sepals [1]. These phenotypes are very similar to the *tag1* mutant in tomato [8] and *agamous-like flower* (*aglf*) mutant in *Medicago truncatula* [16].

The initiation and termination of floral meristem are precisely controlled to ensure the successful development of flowers, in which a set of transcription factors are spatiotemporally coordinated [17]. In *Arabidopsis*, the activity of floral meristem (FM) is maintained through the WUSCHEL-CLAVATA (WUS-CLV) signaling pathway, which plays a key role in maintaining undifferentiated cell populations in the meristem [18, 19]. In addition, the *ag − 2* mutant in *Arabidopsis* produce flowers without stamens and carpels and form indeterminate flowers with reiterating sepals and petals, suggesting AG is very important for floral meristem determinacy [20]. The LEAFY (LFY) gene together with WUSCHEL (WUS) activates *AGAMOUS* (*AG*) at floral stage 3 [21, 22]. While in later stages of the floral development (starting from stage 6), induction of *KNUCKLES* (*KNU*) by *AG* is crucial for the timely termination of FM [23]. In addition, high *AG* level indirectly regulates WUS activity to ensure the proper termination of meristematic activity in the FM, in which a set of regulators including trithorax group protein ULTRAPETALA1 (ULT1), bZIP transcription factor PERIANTHIA (PAN), and other factors such as REBELOTE (RBL) and SQUINT (SQN) are involved [24–28]. The tetramerization of SEPALLATA3 (SEP3) and AG is essential for AG function that activates CRABS CLAW (CRC) and KNU during floral determinacy. In addition, these regulatory networks also interplay with plant hormones during the floral development. It was found that AUXIN RESPONSE FACTOR 3 (ARF3) is transcriptionally regulated by AG and APETALA2 (AP2) in developing flowers, which represses cytokinin activity to inhibit WUS expression [29]. The mechanisms regulating floral development seem to be conserved among species. It has been found that KNU interacts with MINI ZINC FINGER (MIF) to regulate *WUS* expression and this mechanism is conservative between Arabidopsis and tomato [30].

Floral reversion is an unusual process in which the committed floral development is reverted back to vegetative growth, resulting in outgrowth of leaves or inflorescence structures from the first flower [31]. This phenomenon is usually related to varied environmental conditions, such as temperature and photoperiod [31]. For example, the floral reversion was observed in *lfy-6* and *ag-1* mutants of *Arabidopsis* grown in short-day conditions [32]. Floral reversion was also observed in natural allopolyploid *Arabidopsis suecica*, in which abnormal expression of floral genes, including *AGL24*, *APELATA1* (*AP1*), *SHORT VEGETATIVE STAGE* (*SVP*) and *SUPPRESSOR OF CONSTANS1* (*SOC1*) were detected [33, 34]. Unlike in *Arabidopsis*, *LEAFY (LFY)*, *TERMINAL FLOWER1 (TFL1)* and *AG* in *Impatiens balsamina* seemed not to be involved in terminal flowering and floral determinacy [35]. In *Petunia hybrida*, co-suppression of FLORAL BINDING PROTEIN1 (FBP2), a homolog of *Arabidopsis* SEPALLATA-like gene, led to new inflorescences growing from axils of carpels [36]. In tomato, the down-regulated of *TM29*, a homolog of *Arabidopsis* SEPALLATA gene, also resulted in ectopic leafy stems and flowers formed in fruits [37].

In this study, we identified a tomato recessive mutant with the mutation in the gene *Solyc03g006900* which is named *Solanum lycopersicum GT1* (*SlGT11*) based on the previous nomenclature and encodes a transcription factor belonging to Trihelix gene family [38]. Recently, a mutant of *SlGT11* ortholog in *Medicago truncatula* has been reported to control the C-function gene expression and it was named *AGAMOUS-LIKE FLOWER* (*AGLF*) [16]. In *aglf* mutant, the stamens and carpels in the inner whorl are replaced by petals and sepals respectively, resembling the floral phenotype of *ag-1* mutant in *Arabidopsis* [16, 20, 39]. We found that the loss-function of SlGT11 resulted in sepaloid petal at high temperature in the second whorl, carplloid stamen in the third whorl, and ectopic formation of stem-, leaf- and flower-like structures in the fourth whorl. Together with the result that B, C and an E-type MADS-box genes were down-regulated in *slf* mutants, we concluded that *SlGT11* has important functions in the development of the three inner floral whorls. Furthermore, spatiotemporal expression analysis showed that *SlGT11* was expressed throughout the flower in the early stages and its expression became more specific to the primordium position of stamens and carpels in later stages of the floral development. Together our results suggest that *SlGT11* functions in floral organ patterning and maintenance of floral determinacy in tomato.

## Results

### Identification of the *slf* mutant

**Fig. 1:**
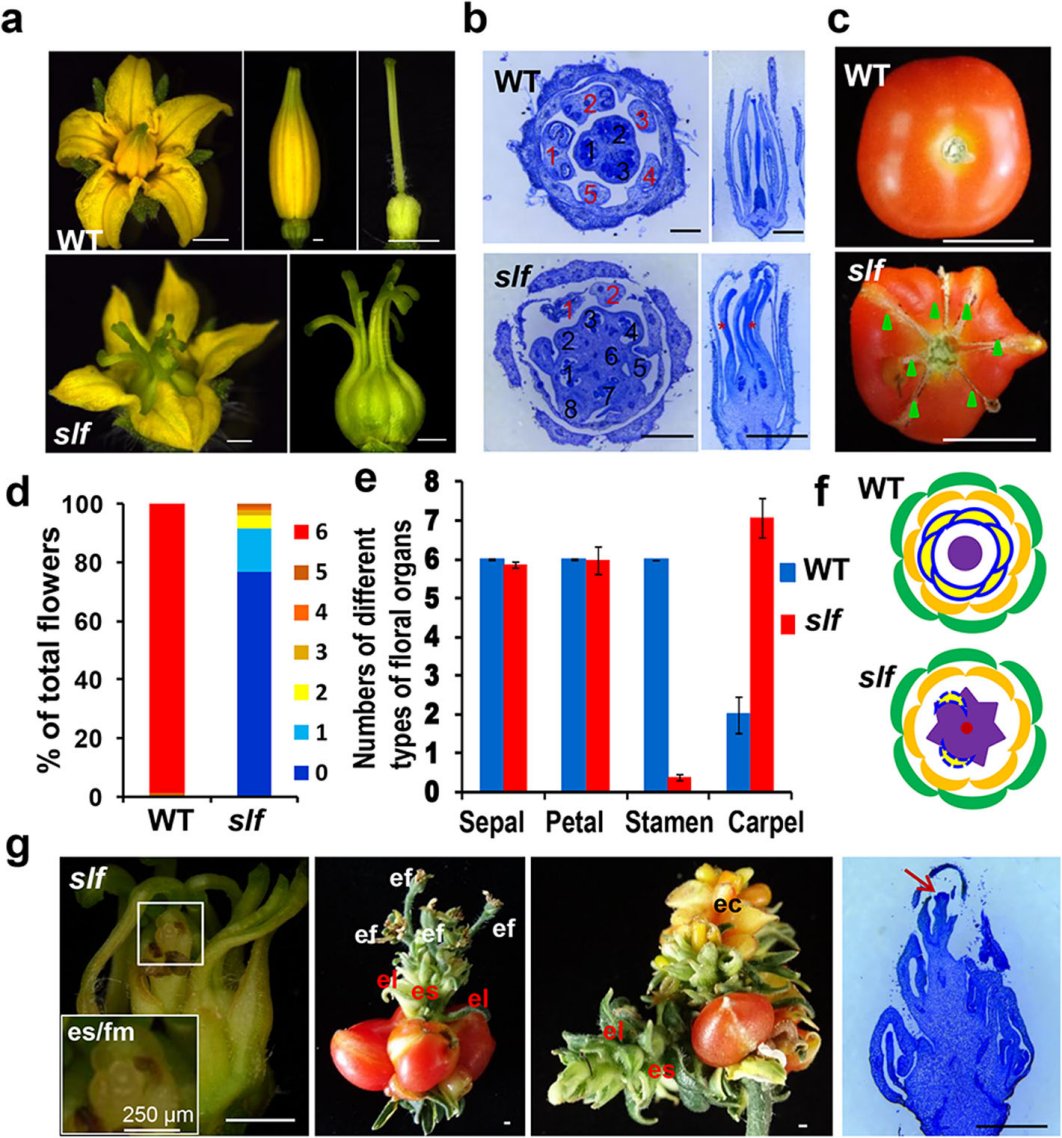
The *slf* mutant shows deficiencies in floral organ identity. **a** The floral phenotypes of WT and *slf.* **b** Transverse and longitudinal sections (stained with toluidine blue) of WT and *slf* flowers at developmental stage 18. Red and black numbers indicate the number of stamens and locules, respectively; red asterisks indicate the conversion of the stamens into pistils in the third whorl. **c** The fruit of WT and *slf*. Green triangles indicate rough radial lines caused by the formation of vestigial stamen-like structures. **d** Percentage of the flowers with 0–6 stamens in WT and *slf*. **e** Quantification of sepals, petals, stamens and carpels in WT and *slf*. The vestigial stamen-like structure in *slf* is counted as the stamen; the carpelloid stamen in *slf* is counted as the carpel. The data represent means ±SD (*n* = 296). **f** A schematic diagram of the floral organs in WT and *slf*. The WT flower consists of four whorls: sepal (green), petal (orange), stamen (yellow and blue solid line), and carpel (purple). The *slf* flower consists of sepals (green) in the 1st whorl, petals (orange) in the 2nd whorl, carpelloid stamens (purple) with or without stunted stamens (yellow and blue dotted line) in the 3rd whorl, and ectopic shoot/floral meristem in the 4th whorl (red circular). **g** Ectopic shoot/floral meristems emerge in the flower, and ectopic shoots produce flowers and leaf-like structures in the fruit, longitudinal sections of the *slf* flowers show ectopic meristem stained with toluidine blue at the floral developmental stage 14. es/fm: ectopic shoot/floral meristem; es: ectopic stem; ef: ectopic flower; el: ectopic leaf; ec: ectopic carpel. Scale bars: (**a**, **b**, **g**) 1 mm

In order to study the mechanism regulating floral organ identity in tomato, we screened tomato EMS-mutant library [40] and identified a mutant (TOMJPG2637–1) with identity defects in stamens and pistils, while the identity and number of sepals and petals were unchanged compared with wild type (WT) **(**Fig. 1a-c, e, g**)**. In the mutant, stamens showed severely carpelloid in the third whorl **(**Fig. 1a**)**. From the longitudinal sections, we verified that the pistil-like structures were formed in the third whorl **(**Fig. 1a, b**)**. Only a few flowers (~ 22.95%) have stamen-like structure remaining in the third whorl of the mutant based on the transverse sections of flowers **(**Fig. 1b, d**)**. The carpelloid stamens of the mutant developed into irregular fruits with more locules and the vestigial stamen structures were later formed radial cracks on the fruit surface **(**Fig. 1c**)**. As this phenotype is similar to previously reported *stamenless* mutant [11, 12], we thus named the mutant *stamenless like flower* (*slf*)*.*

Besides, our histological analyses showed that new shoot/floral meristems instead of carpel primordium formed in the fourth whorl of the mutant **(**Fig. 1. g**)**. The ectopic shoot/floral meristem in the *slf* mutant produced ectopic aberrant foliage and flowers in the fourth whorl, indicating that the normal floral determinacy was lost **(**Fig. 1. g**)**. As a result, the carpelloid stamen in *slf* mutant developed into the parthenocarpic fruit without seeds **(**Fig. 1c, d, Fig. S1c, d**)**. Interestingly, in the carpal-like structure, the ovule development seemed normal in *slf* mutant. Therefore, we attempted to use WT pollen grains for the cross-pollination in *slf* mutant, and only small amount of seeds were obtained. This result may be due to the abnormal pistil-like structures that hindered the pollen-ovule process (Fig. S1e, g). All these results indicated that the *slf* mutant was almost sterile.

### *SlGT11* gene encodes a regulator involved in floral organ identity

**Fig. 2:**
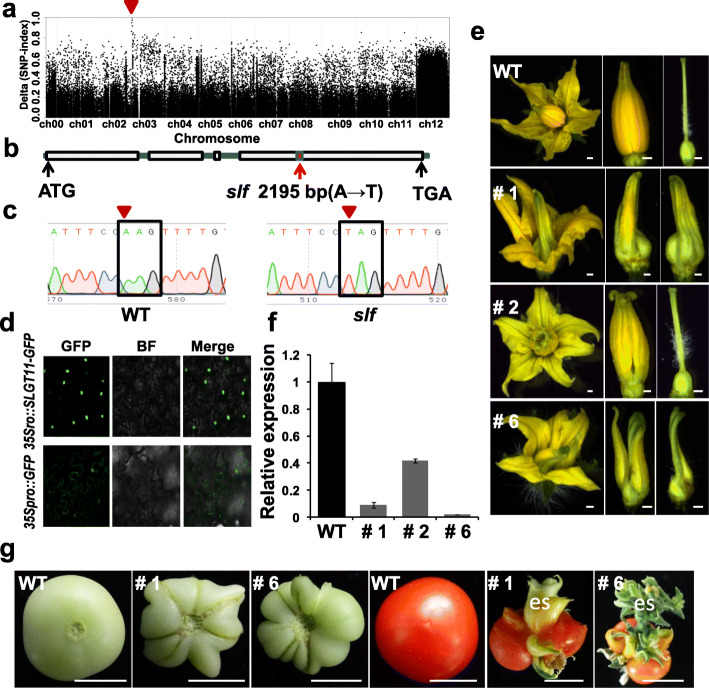
Fine-mapping and functional verification of candidate gene *SlGT11*. **a** The location of *SlGT11* on chromosome 3. Red triangle indicates a signal peak on chromosome 3. **b**, **c** A to T substitution in the position 2195 bp forms a termination codon TAG. **d** Nuclear localization of the SlGT11-GFP fusion protein in tobacco mesophyll cells. **e** Phenotypes of *SlGT11* RNAi transgenic lines (# 1, # 2 and # 6) and WT. **f** qRT-PCR analysis of the *SlGT11* expression in WT, *slf*, and *SlGT11* RNAi lines # 1, # 2, and # 6. *SlACTIN* was used as the internal control. Error bars represent the SD from three biological replicates. **g** The fruits of WT and *SlGT11* RNAi lines # 1 and # 6 show the radial cracks and ectopic stems. #1: *SlGT11*-RNAi-1; #2: *SlGT11*-RNAi-2; #6: *SlGT11*-RNAi-6; Scale bars: **e** 1 mm; **g** 1 cm

To identify the causal gene in *slf* mutant, we first conducted a genetic analysis by crossing the mutant to the WT. In the F2 segregated population, we found 92 progenies resembling the WT and 28 progenies with *slf* phenotypes, which were close to the 3:1 Mendelian segregation rule, indicating that the phenotypes in *slf* were caused by a recessive mutation at a single locus. Through bulked segregant analysis sequencing (BSA-Seq), we identified a signal peak on chromosome 3 **(**Fig. 2a**)**. Further SNP analysis assigned the causal mutation to the gene *Solyc03g006900* which encodes a nucleus-localized Trihelix transcription factor named SlGT11 previously [38], containing a putative GT1 DNA-binding domain and a PKc kinase domain (Fig. S3). The A to T substitution at the 2195 bp position identified forms the termination codon TAG and mRNA level of the *SlGT11* gene in the mutant was significantly decreased **(**Fig. 2b, f**)**. Further sequencing analyses verified that the base substitution occurred in all 28 F2 progenies with *slf* phenotypes **(**Fig. 2c**)**. Subcellular localization in tobacco leaves showed that SlGT11-GFP was located in the nucleus, consistent with the presence of DNA-binding domain **(**Fig. 2d**)**.

To further verify the *SlGT11* function, we transformed WT tomato with an RNA interference (RNAi) plasmid targeting the C-terminus of the *SlGT11*. The phenotypes of 5 independent transgenic RNAi lines were consistent with the *slf* mutant. qRT-PCR verified the significant reduced expression of *SlGT11* in RNAi lines **(**Fig. 2f**)**. The observed phenotypes including carpelloid stamens in the third whorl and new meristem formation from the fourth whorl in RNAi lines (#1 and #6) indicated that *SlGT11* was the gene causing the developmental defects of stamens and carpels in *slf* (Fig. 2e). In addition, abnormal fruits were also found in transgenic lines #1 and #6, indicating that *SlGT11* plays an important role in regulating the floral identity and floral meristem termination (Fig. 2g).

Phylogenetic analysis showed that all the SlGT11 homologous genes in *solanaceae* were grouped into the same cluster, while *Arabidopsis* homologous gene At5g51800 belonged to a less related cluster (Fig. S2). Consistent with this phylogenetic distance, *At5g51800* mutation does not cause the similar floral phenotype, indicating the function of this gene is not completely conserved among different species. The comparative analysis of the amino acid sequence of SlGT11 in *solanaceae* showed that the N-terminal GT1 domain and the C-terminal PKc kinase domain are highly conserved (Fig. S3).

### Spatial and temporal expression pattern of *SlGT11* in tomato

**Fig. 3:**
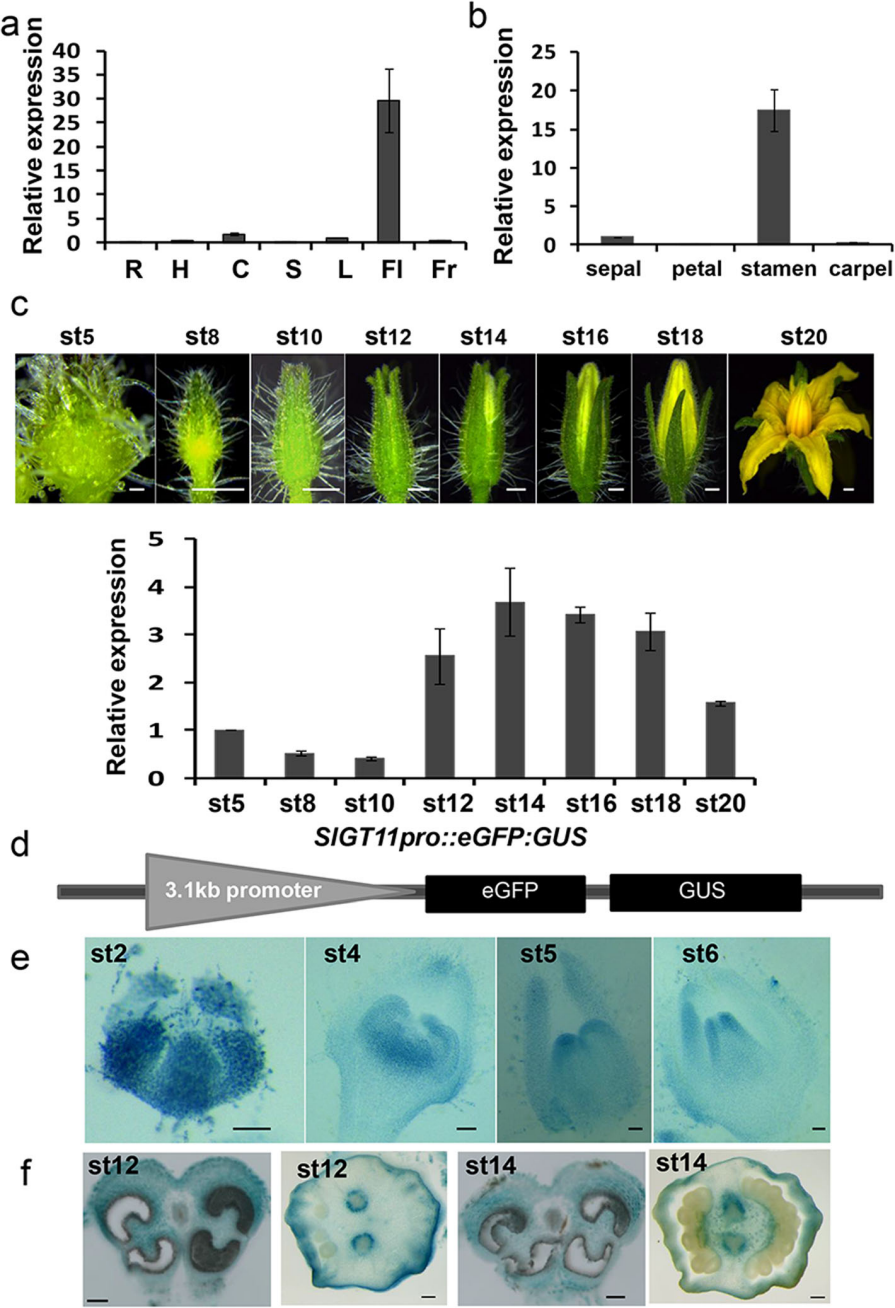
Spatiotemporal expression pattern of *SlGT11.* **a** qRT-PCR analysis of *SlGT11* in different tissues. **b** qRT-PCR analysis of *SlGT11* in different floral organs. **c** qRT-PCR analysis of *SlGT11* at different developmental stages of flower. *SlACTIN* was used as the internal control. Error bars represent the SD from three biological replicates. **d** The diagram of the GUS reporter driven by *SlGT11* promoter. **e** GUS activity was detected throughout the development of the flowers. GUS staining of *SlGT11* promoter was visible in sepals, petals, stamens and carpel primordial, with the strong expression in the stamens and vascular bundles in the ovary. **f** Transverse sections of the stamen and ovary at the stages 12 and 14 showing the GUS activity in anthers and vascular bundles. R: root; H: hypocotyl; C: cotyledon; S: stem; L: leaf; Fl: flower; Fr: fruit; st: stage. Scale bars: (**c** and **e**) 1 mm; (**f**) 100 μm

To examine the expression pattern of *SlGT11*, we performed qRT-PCR in different tomato tissues including roots, hypocotyls, cotyledons, stems, leaves, flowers and fruits. The expression of *SlGT11* was highly enriched in the flowers (Fig. 3a). Then RNA was extracted from different parts of flowers at anthesis for qRT-PCR and we found that *SlGT11* was predominantly expressed in stamens, indicating that *SlGT11* could be important for stamen development (Fig. 3b). Furthermore, we analyzed the temporal expression trend of *SlGT11* during the floral development. qRT-PCR showed that *SlGT11* expression was time-specific, with high expression levels from 6 days to 2 days before flowering (at stage12–18) (Fig. 3c).

Furthermore, we constructed a GUS reporter driven by *SlGT11* promoter and transformed it into WT tomato (Fig. 3d). GUS staining showed that *SlGT11* was expressed throughout the early stages of flowers and the expression became more specific to the stamen and carpel in later stages **(**Fig. 3e**)**. The expression pattern of *SlGT11* in inner two whorls of flower implies that it is probably involved in the regulation of tomato stamen and carpel development.

### Stamen defects occur at the early stage

**Fig. 4:**
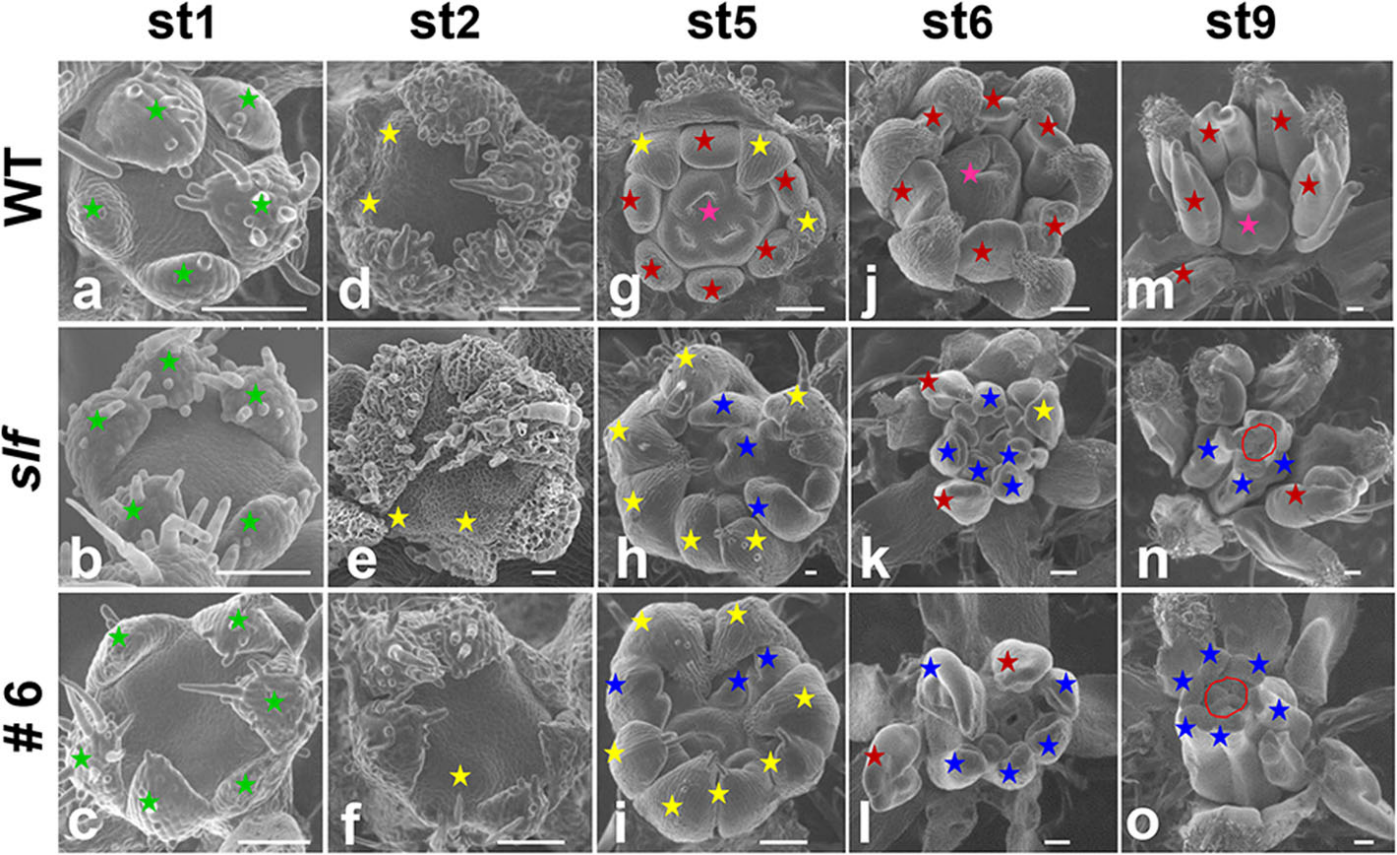
SEM micrographs of floral organs at early developmental stages in WT, *slf* and *SlGT11*RNAi line 6. SEM images of the floral meristem in WT (**a**, **d**, **g**, **j** and **m**), *slf* (**b**, **e**, **h**, **k** and **n**) and *SlGT11*-RNAi-6 (**c**, **f**, **i**, **l** and **o**). Green stars indicate sepal primordia; yellow stars indicate petal primordia; red stars indicate stamen primordia; pink stars indicate carpel primordia; bule stars indicate carpelloid stamens; red lines circle the abnormal carpel primordial with ectopic meristems; Scale bars: 100 μm; st: stage

To investigate how SlGT11 affects stamens and carpels at different floral developmental stages [41], we used scanning electron microscopy (SEM) to visualize the floral development in WT, *slf* and *SlGT11* RNAi line 6 (Fig. 4a-o). The early stages (before the stage 3 when sepal primordia and petal primordia were initiated) of floral development in *slf* and *SlGT11* RNAi line 6 appeared to be similar to that of the WT (Fig. 4a-f). At stage 5, the differences between WT and *slf* or *SlGT11* RNAi line 6 became more prominent. In the WT, six stamen primordia and one carpel primordium with four locules were initiated in the third and fourth whorl respectively (Fig. 4g). In contrast, the third and fourth floral organ primordia in the *slf* and *SlGT11* RNAi line 6 were initiated in disorder (Fig. 4h, j). The defective floral organ identity became more severe in *slf* and *SlGT11* RNAi line 6 at stages 6 and 9 **(**Fig. 4j, m**)**. In the mutant, most stamens were transformed into carpel-like structures, and some ectopic meristems were produced in the central area of the flower **(**Fig. 4k, l, n and o**)**. Combined with the spatiotemporal expression, we concluded that *SlGT11* plays an essential role in the early development of floral organs.

### Expression of floral development genes in *slf* mutant

**Fig. 5:**
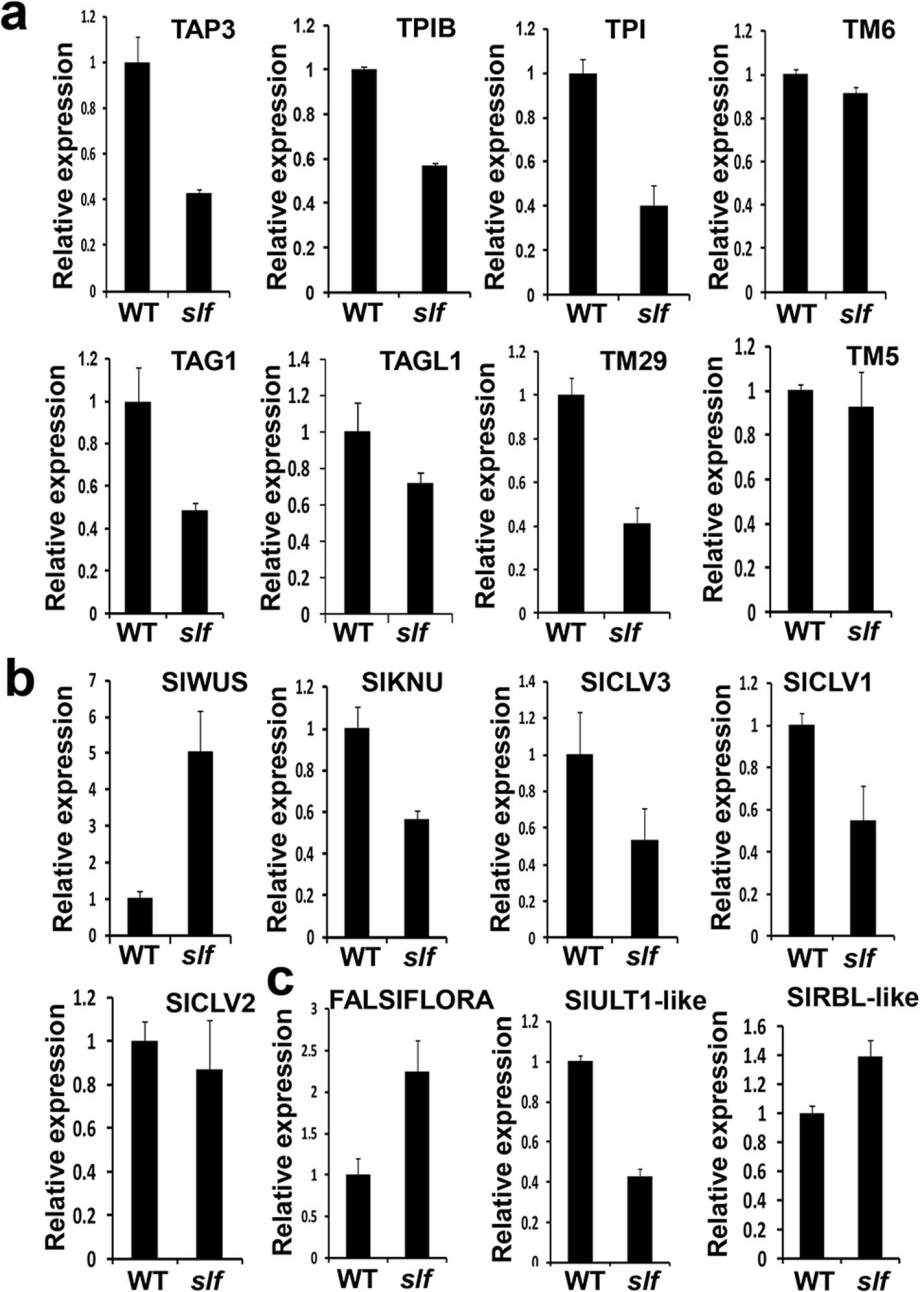
Transcriptional analysis of genes regulating the floral development in WT and *slf.* qRT-PCR analysis of floral organ identity genes *TAP3*, *TPIB*, *TPI*, *TM6*, *TAG1*, *TAGL1*, *TM29* and *TM5* (**a**)*,* meristem maintenance genes *SlWUS*, *SlKNU*, *SlCLV3*, *SlCLV1* and *SlCLV2* (**b**), and floral meristem identity genes *FALSIFLORA, SlULT1-like* and *SlRBL-like* (**c**) in WT and *slf* flowers. *SlACTIN* was used as the internal control

Since the defects of stamens and carpels occurred at the early stages, we compared the expression of BCE genes that were previously reported to affect stamen and carpel identity in the floral buds at stage 1–6 between WT and *slf* [41]. Consistent with the phenotypes, the BCE genes showed the distinct expression pattern between WT and *slf* mutant. Class B genes *TAP3*, *TPI* and *TPIB*, class C genes *TAG1*, *TAGL1* and class E gene *TM29*, were all significantly down-regulated in *slf*. However, the expression level of the B-class gene *TM6* and E class gene *TM5* were not significantly affected in *slf* during the floral development (Fig. 5a).

We next analyzed the expression levels of some regulators involved in floral meristem identity and floral meristem termination. Since the ectopic floral meristem was repeatedly emerged in the later stages of floral development (Fig. 1g), we chose a set of essential genes for floral development including *SlWUS*, *SlKNU*, *SlCLV3*, *SlCLV1*, *SlCLV2*, *FALSIFLORA* (*FA*), *SlULT1-like* and *SlRBL-like* for transcriptional analysis at the later floral stage (stage 20). *FA* and S*lWUS* were up-regulated in *slf*, while *SlKNU*, *SlCLV3*, *SlCLV1* and *SlULT1-like* appeared to be down-regulated in *slf* flowers (Fig. 5b, c). These results were consistent with the floral meristem termination defects in *slf* mutant.

### High temperature inhibits the expression of *SlGT11* and *TM29*

**Fig. 6:**
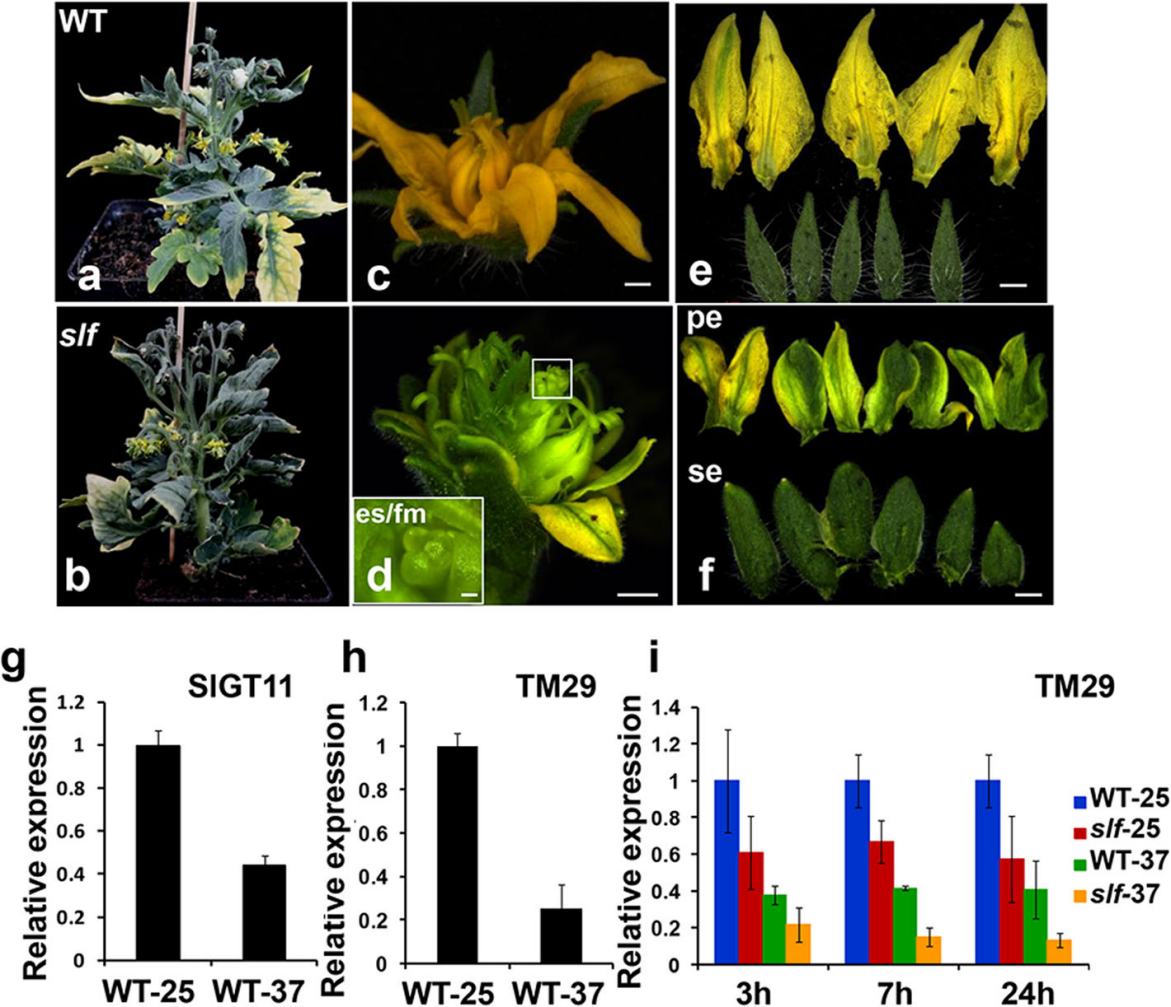
High temperature impacts on the floral identity in WT and *slf* mutant. **a** The flowers of WT treated with high temperature caused stamen separation (**c**), and normal sepals and petals (**e**). **b** The flowers of the *slf* mutant treated with high temperature show greenish petals (**f**) and ectopic shoot/floral meristems (es/fm) in the fourth floral whorl organs (**d**). **g** qRT-PCR analysis of *SlGT11*in WT floral buds treated with 25 °C (WT-25) and 37 °C (WT-37). **h** qRT-PCR analysis of *TM29* in WT floral buds treated with 25 °C (WT-25) and 37 °C (WT-37). (I) qRT-PCR analysis of *TM29* in WT and *slf* mutant floral buds treated with 25 °C (WT-25 and *slf*-25) and 37 °C (WT-37 and *slf*-25) after 3 h, 7 h and 24 h. *SlACTIN* was used as the internal control. Error bars represent the SD from three biological replicates. es/fm, ectopic shhot/floral meristem; se, sepal; pe, petal; Scale bars: (**c**) 1 cm; (**d**) left 250 μm, right 1 mm; (**e**) and (**f**): 1 mm

During the cultivation in the greenhouse where the temperature in summer was higher than the standard, we found that the phenotypes of *slf* became more severe, with stamens hardly visible and the defective flowers with ectopic floral meristem dramatically increased. As the temperature was reported previously to play a role in the floral development [42], we tested whether the *SlGT11* function is also affected by temperature. To that end, we germinated the WT and *slf* mutant seeds at 25 °C for 4 weeks, and grew them in a heated incubator (37 °C daytime/ 28 °C at night) for 20 days. The flowers produced by the *slf* mutant grown at the higher temperature had more carpelloid structures and no stamen-like structures in the third whorl was visible (Fig. 6d). In addition, the petals seemed to partially acquire sepals identity by forming greenish petals with sepal structure (Fig. 6f). Furthermore, we found shoot/floral meristems were produced at the center of the mature flowers (Fig. 6d). Despite the carpelloid stamens and ectopic shoot/floral meristems were also produced in *slf* flowers at lower temperature (25 °C daytime/22 °C night), their occurrence frequency became significantly higher at higher temperature.

To further dissect the influence of the higher temperature on *SlGT11*, we performed qRT-PCR to analyze potential transcriptional change. The floral buds at early stages of WT and *slf* mutant grown at higher temperature were collected for RNA extraction and qRT-PCR. Our results showed that *SlGT11* expression was inhibited by the higher temperature (Fig. 6g). We then examined the expression levels of BCE genes at 3 h, 7 h and 24 h after the high temperature treatment. Our results showed that E class gene *TM29* was further significantly down-regulated by high temperature in *slf* mutant (Fig. 6h, i). Yeast-one-hybrid assay failed to detect the direct binding of SlGT11 to the *TM29* promoter region. All results (Fig. 5**,** Fig. 6 g-i) indicate that SlGT11 indirectly activates *TM29* transcription, and the high temperature further represses the transcription of *SlGT11* and *TM29* both in WT and *slf*.

## Discussion

The classic ABC model was previously established in the model plants *Arabidopsis* and *Antirrhinum majus* [1]. In A-class mutants, flowers have carpels-stamens-stamens-carpels (from the outermost to the innermost whorl), while B-class mutants have sepals-sepals-carpels-carpels flowers and C-class mutants have sepals-petals-petals-sepals flowers. E-class mutants have the flower with all organs resembling sepals [1, 43]. In tomato, the functions of B/C/E class genes seem to be more complicated than those in *Arabidopsis* and *Antirrhinum*. There are four homologous class B genes in tomato: *TAP3*, *TM6*, *TPI* and *TPIB*. Despite similar phenotypes were observed when *TAP3* and *TPIB* were mutated, mutations in *TM6* or *TPI* only resulted in the transformation of stamens into carpels without affecting petals and carpels [6, 7, 13, 44, 45]. Two tomato C class genes TAG1 and TAGL1 have redundant and divergent functions in the floral development [9]. The transgenic plants expressing TAG1 antisense RNA showed homeotic conversion of third whorl stamens into petaloid organs and the emergence of indeterminate floral meristems in the fourth whorl [8]. However, TAGL1 mainly specifies stamens and carpels development in flowers and controls fruit development and ripening [46]. E class gene *TM29* expression was down-regulated by the co-suppression produced aberrant flowers with morphogenetic alterations in the organs of the inner three whorls. In these three whorls, petals and stamens were partially conversed to a sepalloid structure, and ectopic shoots with leaves and secondary flowers emerged from the fruit [37]. In this study, we identified the recessive mutant of *SlGT11* gene whose phenotypes resemble some previously characterized mutants with dysfunctional B/C/E class genes. The carpelloid stamen in *slf* mutants indicates that SlGT11 is required for the function of B type genes in the third whorl. The failed termination of floral meristem and the occurrence of floral reversion in *slf* indicate that the function of C type genes partially requires SlGT11 activity in the fourth whorl. Furthermore, we found that the defects in *slf* were substantially enhanced at higher temperature, with petals transformed into sepals, and the frequency of ectopic shoot/floral meristem in fourth whorl increased. This suggests that SlGT11 is critical in the development of the three inner floral whorls.

*SlGT11* is expressed extensively in the early stage of floral development, but its expression gradually became concentrated in the stamens and the vascular bundles of the ovary. We speculate that *SlGT11* plays the roles in the initiation of each whorl of floral organs, especially the initiation of stamens. It has been reported that the expression of BCE genes which affects stamens development overlaps with *SlGT11* expression domain. Class B genes including *TAP3*, *TM6* and *TPI* were all previously shown to have expression in the stamen position [6, 7]. The C gene *TAG1* is also mainly expressed in the stamens and carpels during the floral development in tomato [8]. Compared with the class B and C genes, the expression of *TM29* at early stage was more extensive, including vascular bundles. But during the later stage of the floral development, *TM29* expression is mainly concentrated in stamens and carpels, which overlaps with the expression domain of *SlGT11* [37]. In addition, the *SlGT11* gene is also expresses in vascular bundles, which could be the origin of the abnormal stem. Compared with the WT, the expression of *TAP3*, *TPI*, *TPIB* and *TM29* in *slf* was all down-regulated, suggesting *SlGT11* could regulate the BCE gene expression to promote the stamens development. Therefore, *SlGT11* could be one of regulators in addition to the ABC model genes that regulate floral organ development.

Floral development is strictly controlled by complex regulatory networks to ensure the successful reproduction of plants. Under natural conditions, the transition from vegetative to reproductive growth is irreversible so the correct tissue patterning can be achieved during the floral development [31]. *slf* mutant has a reversion of floral development to vegetative organs, indicating that meristem termination in flowers becomes defective. As evidenced by a number of previously characterized mutants including *TAP3*, *TPIB* and *TM6*, this reversion phenotype is not necessarily associated with the defects of fused stamens and carpels though [6, 7]. In *Arabidopsis*, the carpels of a weak allele *ag-4* are partially transformed into sepals while the stamens and carpels of a strong allele *ag-6* are completely transformed into petals and sepals [5, 20, 47]. Despite new flowers are formed in the whorl four of *ag-2* flowers, no leaves can be seen, indicating that this defect only represents the aberrant termination of flower meristem [1]. But grown in short day, *ag-1* mutants displayed the reversion of floral meristem back to vegetative development in *Arabidopsis*. Similar phenotype was also reported in *Arabidopsis* mutant *lfy-6* [32]. The direct homologous gene of *AG* in tomato is *TAG1*. In line with the conserved function of *AG*, *tag1* showed homeotic conversion of the third whorl stamens into petaloid organs and the replacement of fourth whorl carpels with indeterminate floral meristems, which are similar to *ag-2* [8]. Transgenic plants expressing *TM29* antisense RNA produced ectopic shoots with partially developed leaves and secondary flowers in the fruit [37]. Here we identified that the inhibition of flower meristem was terminated, and the floral development was reversed into vegetative organs in *slf* mutants, indicating that SlGT11 activity is required for the function of these previously reported genes.

In *Arabidopsis*, stem cell maintenance is lost at the stage 6 of floral development, which makes the flower determinate [48]. In WT flowers, *WUS* mRNA is undetectable at this stage but in *ag* mutants, *WUS* is continuously expressed in the FM, resulting in the disrupted FM termination [48]. In *slf*, *SlWUS* was not repressed in the later stages of tomato floral development. The direct or indirect repressors of *WUS*, such as *SlKNU, TAG1, SlCLVs*, *SlULT1*, were all down- regulated in *slf*. However, the floral meristem identity gene *FA* was up-regulated in *slf,* which was consistent with the defect of floral meristem termination in *slf*.

Interestingly, *AGLF*, the homologous gene of *SlGT11* in *Medicago truncatula*, seemed to function only as the C type gene [16]. Despite the similar expression pattern of *AGLF* and *SlGT11* in the inner two whorls, the different developmental defects of stamens and carpels in respective mutants indicate that this gene likely has the different functions in *Medicago* and tomato. The knockout mutant of the *SlGT11* homologous gene in *Arabidopsis* (*At5g51800*) showed no defectives in floral organ identity [16, 39], indicating that *SlGT11* function may have evolutionarily diverged in different species.

In summary, we found that the loss-function of *SlGT11* resulted in sepaloid petal at high temperatures in the second whorl, carplloid stamen in the third whorl, and ectopic formation of stem-, leaf- and flower-like structures in the fourth whorl. These phenotypes indicate that *SlGT11* has complex functions that are similar to B/C/E-class genes in floral organ specification. Spatiotemporal expression analysis showed that *SlGT11* was expressed throughout the early stages of the floral development, and *SlGT11* expression became more specific to the primordium of stamens and carpels in later stages. Together, our results suggest that *SlGT11* functions in floral organ patterning and maintenance of floral determinacy in tomato.

## Conclusions

The results obtained through this study indicate that the disruption of a novel tomato Trihelix gene *SlGT11* results in the loss of floral organ identity and the reversion of the flower to vegetative development during the floral development. Together with the spatiotemporal expression pattern of *SlGT11*, our results suggest that SlGT11 is essential for the reproductive organ development, but the function of SlGT11 homologous genes is evolutionarily diverged in *Arabidopsis* and *Medicago*. The presented study provides new insight into the function of Trihelix gene SlGT11 in the floral development.

## Methods

### Plant material and growth conditions

All plants used in this study were in tomato (*Solanum lycopersicum L.*) accession Micro-Tom background. Seeds of *stamenless like flower* (*slf*) mutant (TOMJPG2637–1) were obtained from the Tomato Mutants Archive (http://tomatoma.nbrp.jp/). Since the *slf* mutant is partial sterility, seeds from heterozygous plants were used for generating homozygous individuals.

Seeds were pre-germinated on moistened filter paper at 28 °C in complete darkness. Plants were grown under long-day conditions (16-h light/8-h dark) in a greenhouse with a relative humidity of 60%. Daytime and nighttime temperatures were 26 °C and 22 °C, respectively. All plants received regular watering and fertilizer treatments.

### Phenotype characterization

For analyzing the defects of floral organs, we counted the floral organ number of at least 20 flowers on each examined tomato plant. For analyzing the number of stamen in each flower, we collected flowers at anthesis for the quantification. For analyzing the ectopic floral meristem, we removed sepals and petals of examined flowers before anthesis. Immediately after the dissection, morphology of ectopic floral meristem was imaged using Nikon SMZ18 stereomicroscope.

### Histological analysis

To determine morphological and developmental characteristics, fresh floral organs were dissected and examined by Nikon SMZ18 stereomicroscope. The toluidine blue staining was performed as previously described [49]. Briefly the flower buds from the six-week-old WT and *slf* plants were harvested and treated in FAA (3.7% formaldehyde, 5% acetic acid, 50% ethanol) under vacuum conditions for 30 min. These samples were dehydrated in a graded ethanol and tertbutanol series, and then embedded in a paraffin solution containing 50% tertbutanol for 4 h. The infiltrated samples were placed in pure paraffin (Sigma-Aldrich) for over-night.

Sections (10 μm thick) were cut with a Leica RM2255 microtome, and the paraffin was further removed by the dewaxing agent. The tissue parts were washed in pure water carefully, and then stained for 1 min in 0.25% toluidine blue-O (Sigma-Aldrich, U.S.A). All micrographs were photographed with a Nikon SMZ18 stereomicroscope.

### Scanning electron microscopy

Scanning electron microscopy (SEM) analysis of the flowers at the early stage were conducted as following: the sepals and petals were carefully separated from fresh floral organs under stereomicroscope; these samples were observed using a TM3030 PLUS scanning electronic microscopy under a quanta 250 FEG scanning electron microscope at an accelerating voltage of 5 kV.

### Subcellular localization

The 35Spro:SlGT11-GFP and the corresponding empty vector pHellsgate 8 (35Spro:GFP) were transformed into agrobacterium GV3101 and injected into *Nicotiana benthamiana* leaves. The plants with infiltrated leaves were incubated at 25 °C in dark for 24 h and then exposed to light for 12 h before GFP signals were observed by confocal microscopy (LSM 880, Germany Carl Zeiss). The primers were listed in the S1 Table.

### Bulked segregant analysis (BSA)

Bulked segregant analysis was performed according to Chang et al. [50]. The *slf* homozygous plants were used as female parent and crossed to the WT. The F1 plants were then selfed to generate F2 mapping population. For BSA-seq, we extracted genomic DNA from 28 *slf* mutant individuals and 30 WT individuals in the F_2_ mapping population using CTAB method [51]. All DNA quality and concentration were checked before being mixed to construct two bulks (*slf* bulk and WT bulk). The *slf* bulk and WT bulk were sequenced to a depth of 28× and 30× coverage of the tomato genome by HiseqXten-PE150 (Novogene). Trimmed sequences are mapped onto the tomato reference genome (Heinz 1706 cultivar) and mutation variants are filtered. Analysis of the allelic variant frequencies in the pools led to the identification of the causal mutation with 100% frequency in the *slf* bulk. The genes with the expected allelic frequency of 1 were further examined and we conducted transgenic verification for the identified candidate gene. The candidate genes were cloned and sequenced to verify the mutations. The primers were listed in the S1 Table.

### SlGT11 RNAi gene constructs

To generate the SlGT11 RNA interference transgenic plants, we selected a 253 bp-sequence near the 3′ end of the PKc kinase-like domain by Sol Genomics Network vigs tool (https://vigs.solgenomics.net/). The amplified cDNA was cloned into entry vector PDONR221, then further recombined into the binary vector pK7GWIWG2 (II). The binary plasmids were transformed into agrobacterium C58 strain for generating SlGT11 RNAi transgenic lines.

### Plant genetic transformation

Agrobacterium-mediated transformations of tomato were performed according to Brooks et al. [52]. In brief, cotyledon segments from 6- to 8-d old seedlings were precultured for 1d followed by the inoculation with agrobacterium strain C58 containing the RNAi construct. After 2d cocultivation, the cotyledon segments were transferred to a selective regeneration medium containing kanamycin. Subcultures were performed every 15 days until these seedlings produced three true leaves. These seedlings were transferred to a selective rooting medium containing kanamycin. Only well-rooted plants were transferred to the greenhouse.

### Phylogenetic and sequence analyses

Sequences of SlGT11 family members in tomato and other species were obtained from the NCBI database (https://blast.ncbi.nlm.nih.gov/Blast.cgi), and aligned using the ClustalW function in MEGA5. Phylogenetic trees for proteins with 1000 bootstrap replicates were constructed using the maximum likelihood method in MEGA5.

### Imaging, microscopy and GUS staining

To produce the pSlGT11::GUS construct, 3 kb of genomic sequence comprising the SlGT11 upstream region, was cloned into PGWB432 binary vector by the infusion cloning method. Whole floral primordium and flowers at different stages were stained with GUS solution in 37 °C for 10 h after the fixation in cold 90% acetone for 20 min. These samples were dehydrated in a solution (ethanol: acetic acid glacial, in proportions 4:1 by volume) for about 6 h [53]. The samples were then cleared and washed briefly by different concentration ethanol (40% ethanol for 15 min, 20% ethanol for 15 min, 10% ethanol for 15 min). The dehydrated samples were embedded in 5% agar and sectioned by Vibrating slicer (Leica, Germany). The expression pattern of SlGT11 was observed by a Nikon SMZ18 stereomicroscope [54].

### Quantitative real-time PCR analysis

For quantitative real time PCR (qRT-PCR), four-week-olds tomato plants with similar growth conditions were used for tissue collection including roots, hypocotyls, cotyledons, stems, leaves, flowers, fruits, different floral organs and the flowers at different developmental stages [41]. Total RNA was isolated using the Eastep Super Total RNA Extraction Kit (Promega, Shanghai). Subsequently, HIScript II 1st Strand cDNA Synthesis Kit (+gDNA wiper, Vazyme) was used to synthesize the first strand cDNA. ChamQ Universal SYBR qPCR Master Mix kit (Vazyme) was used to perform qRT-PCR reactions in a 7300 Real-Time PCR System (CFX Connect, BIO-RAD). An actin gene was used as the constitutive control. The relative gene expressions were calculated using the 2^inu − ΔΔct^ method [50]. All analyses were performed in three biological replicates and two technical replicates. All primer sequences for qRT-PCR can be found in Table S1.

### Yeast-one-hybrid assay

Yeast-one-hybrid assay was performed using the Matchmaker EYG48 Yeast-one-hybrid system (Clontech) as described by the manual. The coding sequence of SlGT11 for effector protein was cloned into the PJG4–5 vector, and the promoter sequence of B/C/E class genes were cloned into the reporter vector Placzy. Both vectors were transformed into the EYG48 yeast strain. Diploid yeast cells were grown and selected on dropout medium without uracil and tryptophan. To assay protein-promoter interactions, clones were grown on two-dropout medium without uracil and tryptophan, but with x-gal, for 2 d at 30 °C. The empty vectors were used as control. All primer sequences used for cloning can be found in Table S1.

### Yeast-two-hybrid assay

Protein interaction assays in yeast were performed using the Matchmaker Gold Yeast Two-Hybrid System (Clontech) according to the manual. The SlGT11 coding sequence for bait protein was cloned into the pGBKT7 vector and BCE class genes for prey proteins were cloned into the pGADT7 vector. The vectors were then transformed into the Y2HGold yeast strain. Diploid yeast cells were selected and grown on dropout medium without leucine and tryptophan. To assay protein-protein interactions, clones were grown on quadruple-dropout medium without leucine, tryptophan, histidine and adenine for 3 d at 30 °C. All primer sequences used for cloning can be found in Table S1.

## Supplementary Information

**Additional file 1: Figure S1.** Phenotype of ovary and fruit in WT and *slf*.

**Additional file 2: Figure S2.** Phylogenetic analysis of *SlGT11* and its homologs.

**Additional file 3: Figure S3.** Multiple alignment of *SlGT11* and its homologs.

**Additional file 4: Figure S4.** Yeast two-hybrid assays.

**Additional file 5: Figure S5.** Yeast one-hybrid assays.

**Additional file 6: Table S1.** PCR primer sequences used in this study.

## Data Availability

All data generated or analyzed during this study are included in this published article and its supplementary information files. The datasets used and analyzed during the current study are available from the corresponding author on reasonable request.
