# Supplementary figures and images for: SlGT11 controls floral organ patterning and floral determinacy in tomato
Source: BMC Plant Biol. 2020 Dec 14;20:562. doi: 10.1186/s12870-020-02760-2 (PMC7734826; doi:10.1186/s12870-020-02760-2)

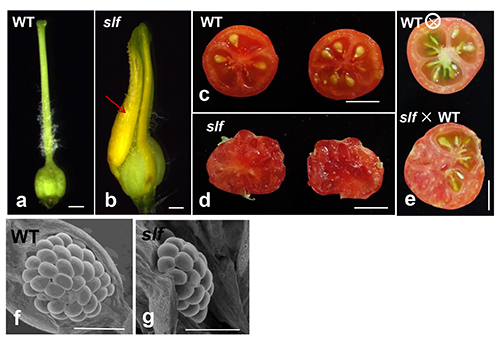

Supplement: Supplementary file 1 — Additional file 1: Figure S1. Phenotype of ovary and fruit in WT and slf. [file 12870_2020_2760_MOESM1_ESM.tif]

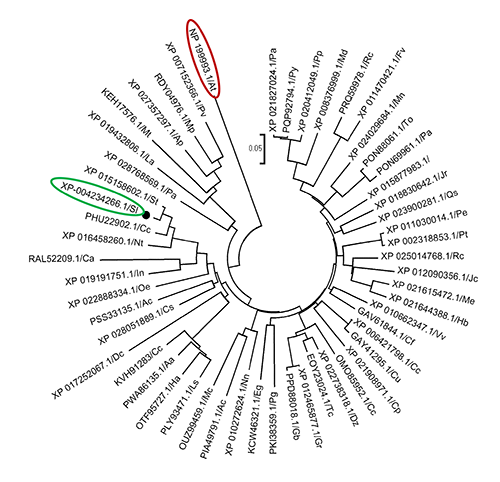

Supplement: Supplementary file 2 — Additional file 2: Figure S2. Phylogenetic analysis of SlGT11 and its homologs. [file 12870_2020_2760_MOESM2_ESM.tif]

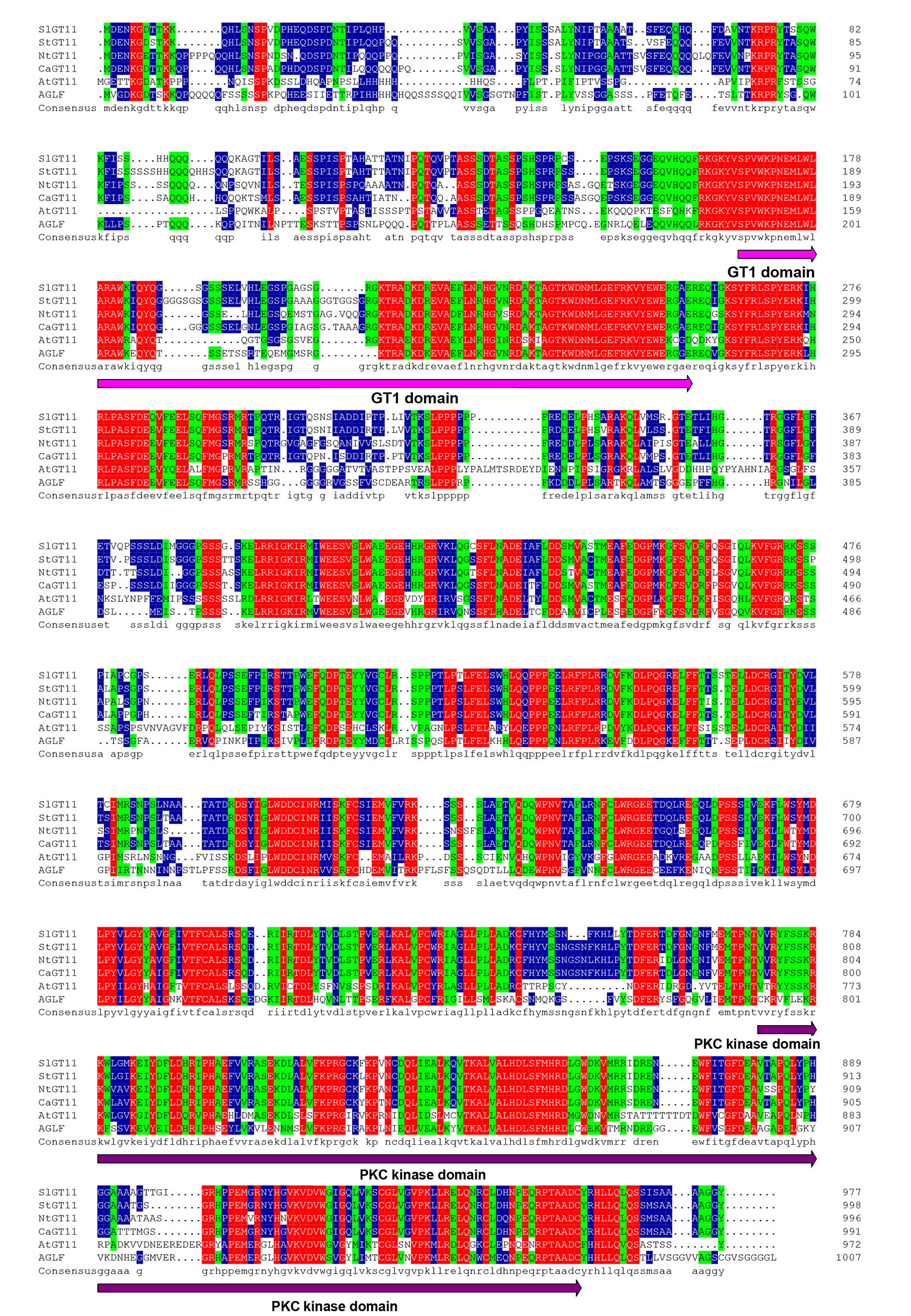

Supplement: Supplementary file 3 — Additional file 3: Figure S3. Multiple alignment of SlGT11 and its homologs. [file 12870_2020_2760_MOESM3_ESM.tif]

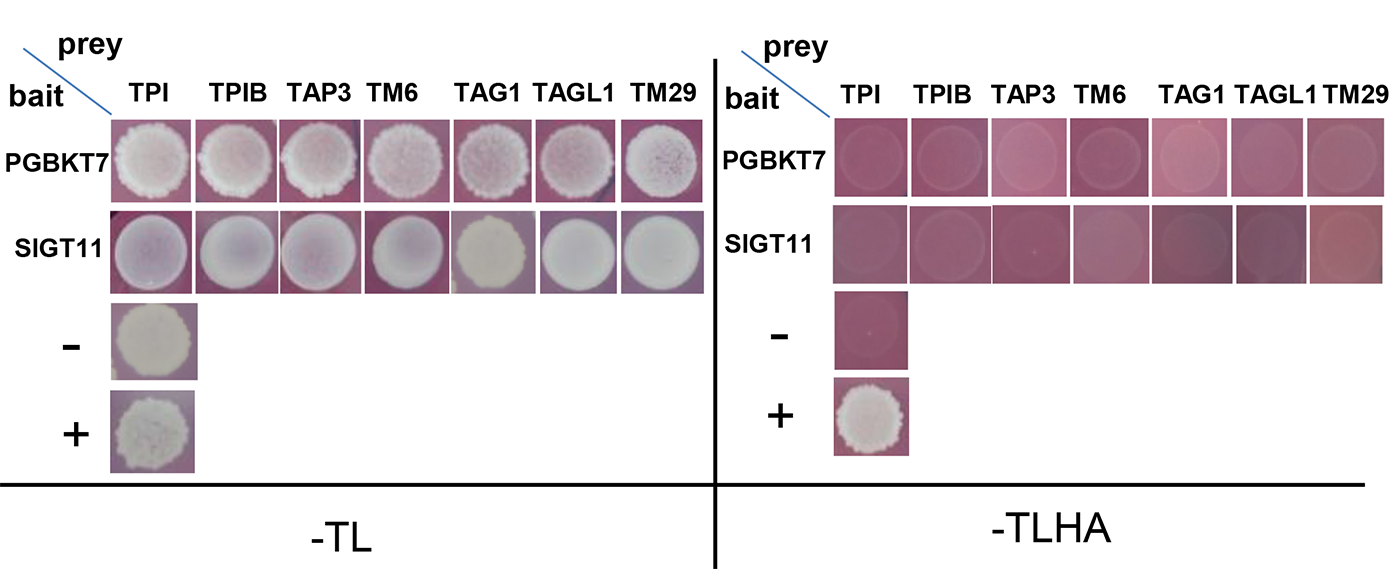

Supplement: Supplementary file 4 — Additional file 4: Figure S4. Yeast two-hybrid assays. [file 12870_2020_2760_MOESM4_ESM.tif]

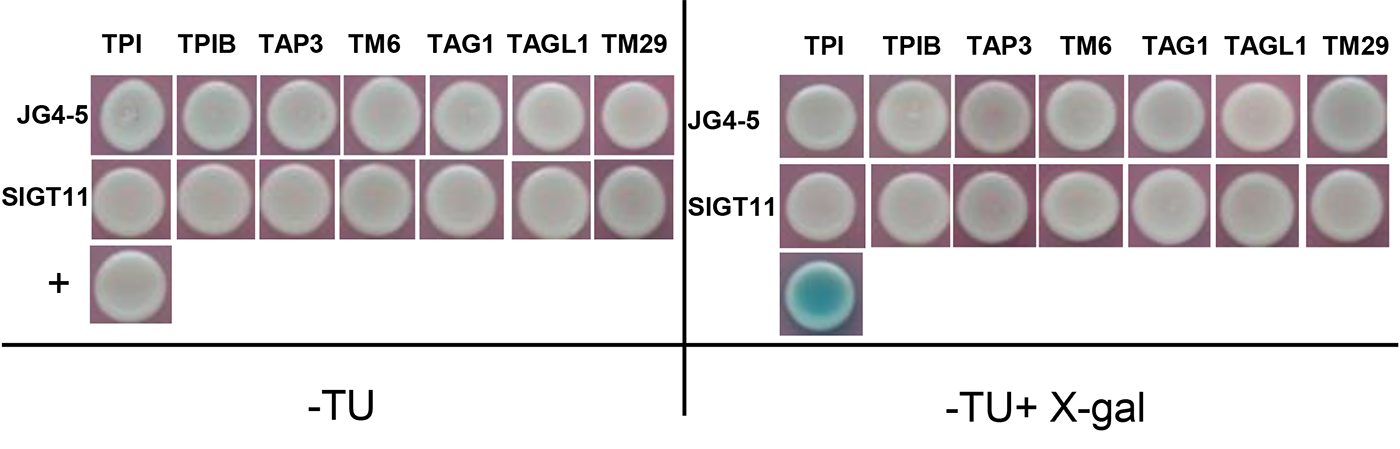

Supplement: Supplementary file 5 — Additional file 5: Figure S5. Yeast one-hybrid assays. [file 12870_2020_2760_MOESM5_ESM.tif]
